# Editorial: Glial and Neural Stem Cells as New Therapeutic Targets for Neurodegenerative Disorders

**DOI:** 10.3389/fncel.2020.00071

**Published:** 2020-04-03

**Authors:** Sara Xapelli, Maria J. Diógenes, Vincenzo Crunelli, Carlos P. Fitzsimons, Sandra H. Vaz

**Affiliations:** ^1^Instituto de Medicina Molecular João Lobo Antunes, Faculdade de Medicina, Universidade de Lisboa, Lisbon, Portugal; ^2^Instituto de Farmacologia e Neurociências, Faculdade de Medicina, Universidade de Lisboa, Lisbon, Portugal; ^3^Neuroscience Division, School of Bioscience, Cardiff University, Cardiff, United Kingdom; ^4^Department of Physiology and Biochemistry, Malta University, Msida, Malta; ^5^Swammerdam Institute for Life Sciences, Center for Neuroscience, University of Amsterdam, Amsterdam, Netherlands

**Keywords:** neural stem/progenitor cells, glial cells, astrocytes, microglia, neurodegenenerative diseases

The gradual and progressive degeneration of neural cells is a common feature of different neurological disorders culminating in a disturbed neuronal communication. The remarkable discovery that adult brain has the capacity to develop new neurons, as well as new glial cells, raised the hope to restore neuronal communication. It is now accepted that adult neurogenesis occurs in neurogenic niches which are under the control of several factors including neurotrophins. These neurogenic niches contain multipotent neural stem/progenitor cells (NSPCs) which can differentiate into neurons, astrocytes and oligodendrocytes. Interestingly, different brain injuries or pathologies, such as epilepsy, head traumas and stroke, induce neurogenesis and/or astrogliogenesis. This increase in neurogenesis has been proposed as an endogenous attempt to repair and reduce brain damage. On the contrary, aging and several neurodegenerative disorders promote a decrease in neurogenesis. Nevertheless, NSPC-based therapy is, to date, not yet a satisfactory strategy due to poor survival and differentiation levels, as well as reduced synaptic plasticity either after transplantation or neural injury. Therefore, it is important to study the molecular and cellular mechanisms of NSPC's function in detail, and their potential therapeutic applicability to improve long-term survival, differentiation, and synaptic integration of derived newborn neural cells.

Classically, the studies in neurodegenerative disorders are mainly focused on neuronal abnormalities, while the non-neuronal cells were almost neglected. Nevertheless, there is increasing evidence that glial cells, and in particularly astrocytes and microglia, are very important in the pathology of these disorders. Astrocytes play important roles in neuronal synaptic activity by removing the majority of glutamate present at the synapse and also by releasing modulators/transmitters that interact with neuronal receptors. Moreover, in pathological conditions astrocytes have been implicated in the onset and progression of several diseases with reactive astrogliosis a common feature. In many neurodegenerative disorders, several changes in astrocytic function have been detected, namely in calcium signaling and in the release of inflammatory molecules. Furthermore, microglia plays an important role in managing neuronal cell death, neurogenesis, and synaptic interactions, besides their involvement in immune-response generating cytokines. Most likely astrocytes and microglia are possible candidates as new pharmacological targets that can ameliorate some of the features of neurological disorders.

This Research Topic containing 10 manuscripts highlights the role of glial and NSPCs in physiological and pathological conditions. La Rosa et al. showed that enhancing the expression and activity of the antioxidant response the master regulator nuclear factor erythroid 2–related factor 2 ameliorates the phenotypic defects observed in NSPCs, re-establishing a proper differentiation program. Lauro et al. reported that the chemokine CX3CL1 (fractalkine) protects against cerebral ischemia modulating the activation state of microglia and its metabolism in order to restrain inflammation and organize a neuroprotective response against the ischemic insult. The mini-review by Peteri et al. discussed that species-specificity of astrocytes poses a challenge in translating results obtained from animal studies to humans, and patient-derived induced pluripotent stem cells offer an alternative to disease modeling. The data described by Pereira de Melo Guimarães et al. revealed that neurogenin 2 induces lineage conversion of postnatal rodent müller glia cells into retinal ganglion cell-like induced neurons *in vitro* and resumes the generation of this neuronal type from late progenitors of the retina *in vivo*. Ferreira et al. described an interaction between brain-derived neurotrophic factor and endocannabinoid signaling in the control of neurogenesis at distinct levels, further contributing to highlight novel mechanisms in the emerging field of brain repair. In the review by Casares-Crespo et al. the role of basal autophagy in adult neural stem cells and neurogenesis was discussed. Bribian et al. detailed the clonal glial response in a multiple sclerosis mouse model. Pan et al. provided data showing that small molecule (Methyl 3,4-dihydroxybenzoate) promotes NSCs differentiation into cholinergic motor neurons by regulating cell cycle and the cholinergic neuronal differentiation Isl1 gene. Zhang et al. reviewed the relationship between microglia and depression, elucidating the mechanism by which microglia regulate inflammation and neurogenesis in response to major depression disorder. Gonzalez-Perez et al. showed that tactile inputs from vibrissal follicles strongly modify the expression of c-Fos and calbindin in the dentate gyrus, disrupt different aspects of hippocampal neurogenesis, and support the notion that spatial memory and navigation skills strongly require tactile information in the hippocampus.

Overall, the outstanding manuscripts including both reviews and original articles of this Research Topic provide evidence for a role of glial cells, namely microglia and astrocytes, and NSPCs in physiological and pathological conditions. The study of glial cells and NSPCs will continue to reveal novel and effective therapeutic mechanisms for disorders of the nervous system. We hope that this collection of articles will be highly attractive to the neuroscience community.

## Author Contributions

All authors listed have made a substantial, direct and intellectual contribution to the work, and approved it for publication.

### Conflict of Interest

The authors declare that the research was conducted in the absence of any commercial or financial relationships that could be construed as a potential conflict of interest.

